# Neuroprotective Roles of l-Cysteine in Attenuating Early Brain Injury and Improving Synaptic Density *via* the CBS/H_2_S Pathway Following Subarachnoid Hemorrhage in Rats

**DOI:** 10.3389/fneur.2017.00176

**Published:** 2017-05-02

**Authors:** Tong Li, Lingxiao Wang, Quan Hu, Song Liu, Xuemei Bai, Yunkai Xie, Tiantian Zhang, Shishi Bo, Xiangqian Gao, Shuhua Wu, Gang Li, Zhen Wang

**Affiliations:** ^1^Department of Neurosurgery, Qilu Hospital of Shandong University and Brain Science Research Institute, Shandong University, Jinan, China; ^2^Department of Physiology, Shandong University School of Medicine, Jinan, China; ^3^Department of Neurosurgery, Taian Central Hospital, Taian, China; ^4^Department of Pathology, Binzhou Medical University Hospital, Binzhou, China

**Keywords:** l-cysteine, H_2_S, cystathionine-β-synthase, subarachnoid hemorrhage, early brain injury

## Abstract

l-Cysteine is a semi-essential amino acid and substrate for cystathionine-β-synthase (CBS) in the central nervous system. We previously reported that NaHS, an H_2_S donor, significantly alleviated brain damage after subarachnoid hemorrhage (SAH) in rats. However, the potential therapeutic value of l-cysteine and the molecular mechanism supporting these beneficial effects have not been determined. This study was designed to investigate whether l-cysteine could attenuate early brain injury following SAH and improve synaptic function by releasing endogenous H_2_S. Male Wistar rats were subjected to SAH induced by cisterna magna blood injection, and l-cysteine was intracerebroventricularly administered 30 min after SAH induction. Treatment with l-cysteine stimulated CBS activity in the prefrontal cortex (PFC) and H_2_S production. Moreover, l-cysteine treatment significantly ameliorated brain edema, improved neurobehavioral function, and attenuated neuronal cell death in the PFC; these effects were associated with a decrease in the Bax/Bcl-2 ratio and the suppression of caspase-3 activation 48 h after SAH. Furthermore, l-cysteine treatment activated the CREB–brain-derived neurotrophic factor (BDNF) pathway and intensified synaptic density by regulating synapse proteins 48 h after SAH. Importantly, all the beneficial effects of l-cysteine in SAH were abrogated by amino-oxyacetic acid, a CBS inhibitor. Based on these findings, l-cysteine may play a neuroprotective role in SAH by inhibiting cell apoptosis, upregulating CREB–BDNF expression, and promoting synaptic structure *via* the CBS/H_2_S pathway.

## Introduction

In patients with subarachnoid hemorrhage (SAH), early brain injury (EBI) is the primary cause of high mortality and morbidity (1). Multiple factors, including cell death, oxidative stress, abnormal inflammatory responses, and cerebral vasospasm, are involved in the mechanisms underlying EBI after SAH (2). Thus, identification of early neuroprotective strategies for potential clinical use is urgently needed.

l-Cysteine is a semi-essential amino acid and is important for regulating human metabolism (3). The three traditional endogenous sources of l-cysteine include absorption from the diet, the transsulfuration pathway, and protein degradation. Disruption of the extracellular l-cysteine/l-cystine ratio may be associated with oxidative stress (4, 5). Kimura et al. demonstrated that in the central nervous system (CNS), l-cysteine may be catalyzed by cystathionine-β-synthase (CBS), which is expressed in astrocytes, and may then produce endogenous hydrogen sulfide (H_2_S) (6, 7). Moreover, amino-oxyacetic acid (AOAA), a widely used selective CBS inhibitor, has been reported to block CBS-mediated H_2_S production in several organs (7, 8).

H_2_S plays multiple roles in the CNS under both physiological and pathological conditions (9). Interestingly, accumulating evidence has suggested that exogenous H_2_S can function as a powerful neuroprotective agent. Kimura and Kimura reported in 2004 that H_2_S protected primary rat cortical neurons from oxidative stress-induced injury (10). H_2_S also exerts a number of cytoprotective anti-apoptotic, antioxidant, and anti-inflammatory effects on the CNS (6, 11, 12). Our previous studies showed that H_2_S exhibited neuroprotective potential in an animal model of cerebral hypoxia injury (13, 14). Importantly, we observed that l-cysteine promoted the proliferation and neuronal differentiation of neural stem cells *via* the CBS/H_2_S system *in vitro* (15). Administering AOAA to animal models of cerebral hypoxia injury could inhibit H_2_S generation and induce physiological changes in blood pressure regulation or associative learning (7, 16).

Only limited information is available about the neuroprotective effects of H_2_S on SAH (17, 18). Furthermore, whether l-cysteine can safely exert protective effects on EBI after SAH by triggering CBS to produce H_2_S and the molecular mechanisms underlying these effects are still unknown. Thus, the aim of this study is to elucidate the potential therapeutic effect of l-cysteine on EBI after SAH and determine whether l-cysteine is associated with H_2_S function.

## Animals and Methods

### Animals

Male Wistar rats (280–350 g) were purchased from the Laboratory Animal Center, Shandong University. Upon arrival, the animals were housed under standard laboratory conditions (temperature 20 ± 2°C, 12 h:12 h light/dark cycle, lights on at 0800 h), provided free access to food and water and allowed to habituate to their new environment for 1 week.

### SAH Model

Experimental SAH was induced in the rats using double blood injection according to our previous study (18). Briefly, esthesia was induced under 3.5% isoflurane and changed to continuous narcosis with 2.5% isoflurane during surgery. A catheter was inserted into the femoral artery under sterile conditions to withdraw blood and measure blood pressure. Two hundred microliters of autologous blood was withdrawn from the femoral artery and injected into the cisterna magna over a 3-min period.

### Experimental Design

A total of 134 surgeries were conducted. The rats were randomly assigned to the following five groups: Sham (*n* = 22), Sham + l-cysteine (*n* = 22), SAH (*n* = 30), SAH + l-cysteine (*n* = 30), and SAH + l-cysteine + AOAA (*n* = 30). At 48 h after SAH, these rats were euthanized, and the prefrontal cortex (PFC) tissues were removed and prepared for analysis. The individual group mortality within 48 h after surgery was as follows: Sham 0% (0/22), Sham + l-cysteine 0% (0/22), SAH 23.3% (7/30), SAH + l-cysteine 10% (3/30), and SAH + l-cysteine + AOAA 16.7% (5/30).

### Drug Administration

l-Cysteine (Sigma-Aldrich) was dissolved in vehicle (PBS) at a working concentration of 100 mM as determined by our previous research (15), and 30 µL of the l-cysteine solution was intracerebroventricularly administered 30 min after SAH. AOAA (Sigma-Aldrich) was dissolved in vehicle (PBS), and a 5 mg/kg dose was intraperitoneally administered with l-cysteine.

### CBS Activity Assay and Measurement of H_2_S Production

The CBS activity of brain tissue was detected by a CBS assay kit (Genmed Scientifics Inc., China). This assay indirectly measures CBS activity by detecting CBS metabolites that interact with NADPH. Absorbance was measured at 340 nm using a microplate reader (Spectra Max 190, Molecular Devices, Sunnyvale, CA, USA).

To quantify H_2_S, we used the traditional methylene blue method. Briefly, the PFC tissue was homogenized and incubated with zinc acetate, which generates zinc sulfide that subsequently reacts with *N*,*N*-dimethyl-*p*-phenylenediamine sulfate (NNDPD). The absorbance value was determined at 670 nm, and the H_2_S level was calculated against an NaHS calibration curve.

### Neurological Scores

At 48 h after SAH, neurological function was evaluated by two “blinded” investigators using a modified Garcia scoring system (19, 20). This system comprises the following seven subtests: spontaneous activity (0–3 points), reaction to side stroking (1–3 points) and to vibrissae touch (1–3 points), limb symmetry (0–3 points), forelimb outstretching (0–3 points), and climbing (0–3 points) and beam walking (0–4 points) abilities. The total score of these subtests reflected neurological function. High Garcia scores indicated better neurological function, and low scores indicated worse function, with the worst performances receiving 2 points (21).

### Brain Water Content

Brain edema was determined according to the wet/dry method, where% brain water content = [(wet weight − dry weight)/wet weight] × 100%. Briefly, each brain sample (both cerebral hemispheres) was removed from the skull and weighed immediately. Then, the sample was dried at 100°C for 48 h and weighed to determine the dry weight.

### Hematoxylin and Eosin Staining

Animals were perfused under deep anesthesia with 10% chloral hydrate followed by 4% paraformaldehyde. The brains were then removed and post-fixed in formalin. After fixation and dehydration in an ethanol gradient, the brain tissue was embedded in paraffin and sliced into 4-μm thick coronal sections using a section cutter (Leica, Germany). The sections (3 sections/rat) were stained with hematoxylin and eosin (H&E). In addition, four rats in each group were prepared for H&E staining. The morphology of the PFC (the cerebral cortex that covers the anterior portion of the frontal lobe) was observed under a light microscope (Olympus Corporation, Japan).

### Transferase dUTP Nick End Labeling (TUNEL) Staining

Four samples from each group were prepared for terminal deoxynucleotidyl TUNEL staining. Apoptosis was detected using a TUNEL kit according to the manufacturer’s protocol (DeadEnd Fluorometric kit, Promega, WI, USA). Slides were then counterstained with 4′,6-diamidino-2-phenylindole (DAPI), washed, coverslipped with a water-based mounting medium, and sealed with nail polish. Three microscope fields (20×) containing TUNEL-positive cells in the cortex were selected and imaged. The number of TUNEL/DAPI-positive cells was calculated as the mean of the numbers obtained from six images per rat. Counting was performed in a blinded manner.

### Immunofluorescence Imaging

Slides (*n* = 4 samples per group) were fixed in 4% paraformaldehyde for 20 min and blocked with 10% goat serum in PBS. The slides were subsequently incubated overnight in a humidified chamber at 4°C with the following primary antibodies: NeuN (1:100, Abcam, Cambridge, MA, USA) and cleaved caspase-3 (1:100, Cell Signaling Tech., MA, USA). After primary antibody incubation, the samples were washed and incubated with an appropriate fluorescent-conjugated secondary antibody (1:500 dilution, Sigma-Aldrich) for 1 h. Images were captured using a Nikon TE2000U microscope. Three microscope fields (20×) containing cleaved caspase-3/NeuN double-positive cells in the cortex were chosen and imaged. The number of active caspase-3/NeuN double-positive cells was calculated as the mean of the numbers obtained from six images per rat. Counting was performed in a blinded manner.

### Immunohistochemistry

The sections were deparaffinized using a standard procedure and washed with PBS as described previously. Briefly, after blocking for 30 min at room temperature, the sections were incubated with the following primary antibodies: CBS (1:200, Santa Cruz Biotechnology, Santa Cruz, CA, USA) at 4°C overnight. After primary antibody incubation, the samples were washed and incubated with secondary antibodies for 2 h at room temperature. The sections were washed and then incubated with an avidin–biotinylated enzyme complex for 1 h at room temperature. The sections were visualized with diaminobenzidine. Nuclei were counterstained with hematoxylin. Finally, the sections were dehydrated in an alcohol gradient and cleared with xylene. Images were captured using a Nikon TE2000U microscope.

### Sample Preparation for Transmission Electron Microscopy (TEM)

For TEM, we sacrificed three rats per group. PFC specimens, with an approximate volume of 1 mm^3^, were dissected quickly on ice and fixed in 2.5% glutaraldehyde for 2 h at 4°C. Following several washes in PBS, the specimens were fixed in 1% osmium tetroxide for 2 h and then dehydrated in a graded ethanol series. The tissues were subsequently infiltrated with 50/50 propylene oxide overnight and embedded. The tissues were prepared for sectioning on an Ultramicrotome (EM UC 7, Leica, Germany) and cut into 50-nm thick sections. After being stained with uranyl acetate, the sections were examined under a Hitachi H-7500 TEM.

### Reverse Transcription Polymerase Chain Reaction (RT-PCR)

Total RNA was extracted from the PFC using a TRIzol reagent (Gibco, Invitrogen) according to the manufacturer’s instructions. The RNA concentration was determined using a spectrophotometer (Bio-Rad Labs) at 260 nm. Identical amounts of RNA (2 µg) were reverse transcribed into cDNA using a commercial RT-PCR kit (Fermentas, Vilnius, Lithuania) according to the manufacturer’s instructions. Then, the cDNA was subsequently amplified by PCR with specific primers (Table 1). The PCR products, which were separated on a 1.2% agarose/TAE gel, were visualized by staining with ethidium bromide. The densitometric values were normalized to those of β-actin. Band intensity was determined using Image-Pro Plus 6.0 software.

**Table 1:** **PCR primers used in this study**.

Gene	Forward (5′→3′)	Reverse (5′→3′)
Bax	GGT TGC CCT CTT CTA CTT TGC	TCT TCC AGA TGG TGA GCG AG
Bcl-2	GGA TGA CTT CTC TCG TCG CTA C	TGA CAT CTC CCT GTT GAC GCT
Brain-derived neurotrophic factor	AGC TGA GCG TGT GTG ACA GT	ACC CAT GGG ATT ACA CTT GG
Synaptophysin	CAAGAAATACCGCTACCAAGATG	CCCTCTGTTCCATTCACCTG
PSD95	ATGGCACGTAATGGAGACTAC	TCTTGTGTAGTCGAACCATCTG
β-actin	CTA TTG GCA ACG AGC GGT TCC	CAG CAC TGT GTT GGC ATA GAG G

### Western Blot Analysis

Protein concentration in the PFC was determined using a BCA protein assay kit (Pierce Biotechnology, Inc.). A quantity of 30–50 µg of total proteins was loaded onto a 4–20% gradient polyacrylamide gel, electrophoretically transferred to a polyvinylidene difluoride membrane and probed with the following primary antibodies: Bax antibody (1:1,000, Santa Cruz Biotechnology, CA, USA), Bcl-2 antibody (1:1,000, Santa Cruz Biotechnology), cleaved caspase-3 (1:500, Cell Signaling Tech. MA, USA), caspase-3 (1:1,000, Cell Signaling), phospho-cAMP response element binding protein (p-CREB) (1:1,000, Cell Signaling Tech., MA, USA), CREB (1:1,000, Cell Signaling Tech., MA, USA), and brain-derived neurotrophic factor (BDNF) (1:1,000, Santa Cruz Biotechnology). β-actin (1:2,000; Sigma-Aldrich) was used as an internal control. The secondary antibody was horseradish peroxidase conjugated to goat/mouse anti-rabbit IgG (1:8,000, Sigma-Aldrich). The membranes were developed using an enhanced chemiluminescence detection system (Pierce, Rockford, IL, USA).

### Statistical Analysis

SPSS 22.0 was used for statistical analysis. The neurological scores were analyzed by Kruskal–Wallis one-way analysis of variance (ANOVA) on ranks followed by Dunn’s *post hoc* test. Other data are presented as the mean ± SD; these data were analyzed by one-way ANOVA followed by Tukey’s *post hoc* analysis. Differences were considered significant at *p* < 0.05.

## Results

### The Effect of l-Cysteine on CBS Activity and H_2_S Production in SAH-Insulted Brain Tissue

**Figure 1:**
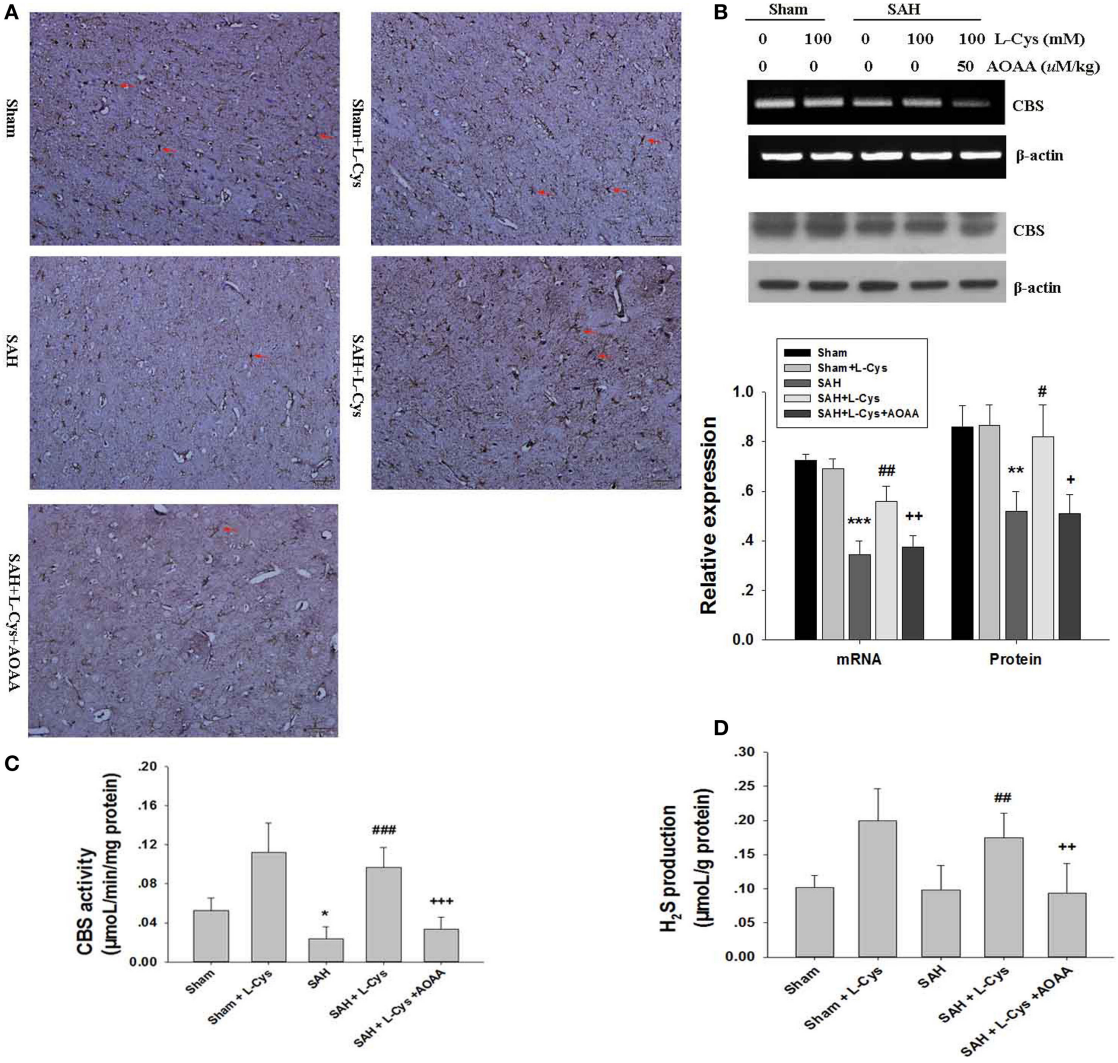
**Effects of l-cysteine on endogenous cystathionine-β-synthase (CBS) activity**. **(A)** The expression of CBS in cells (red arrows indicated) was determined by immunohistochemistry at 48 h after subarachnoid hemorrhage (SAH). Scale bar = 50 μm (*n* = 4). **(B)** CBS was quantified by reverse transcription polymerase chain reaction and Western blotting at 48 h after SAH. Each value was normalized to β-actin. The bar graphs showing the quantification of mRNA and protein levels of CBS were generated by Image-Pro Plus 6.0 (*n* = 4). **(C)** CBS activity was assessed at 48 h after SAH (*n* = 6). **(D)** Production of endogenous H_2_S was evaluated by the methylene blue method at 48 h after SAH (*n* = 8). The values represent the means ± SD. *p < 0.05, **p < 0.01, ***p < 0.001 SAH vs Sham, ^#^*p* < 0.05, ^##^*p* < 0.05, ^###^*p* < 0.001 SAH + l-Cys vs SAH, ^+^*p* < 0.05, ^++^*p* < 0.01, ^+++^*p* < 0.001 SAH + l-Cys + AOAA vs SAH + l-Cys. AOAA, amino-oxyacetic acid.

Cystathionine-β-synthase has been reported to mainly localize to astrocytes in the CNS and catalyze l-cysteine to produce endogenous H_2_S (22, 23). Here, we investigated SAH-induced changes in CBS expression and CBS activity in response to l-cysteine treatment at 48 h after SAH. In agreement with previous findings, our immunohistochemical analysis revealed numerous CBS-positive cells in the PFC tissue of the Sham and Sham + l-cysteine groups, whereas CBS-positive cells were very rare in the SAH group (Figure 1A). Surprisingly, l-cysteine treatment significantly upregulated CBS expression in the SAH group (Figure 1A). Moreover, CBS expression in the PFC was evaluated at 48 h after SAH by Western blot and RT-PCR, and the results revealed that l-cysteine also increased the protein and mRNA expression levels of CBS (Figure 1B).

We further assessed CBS activity, which affects l-cysteine. Both the Sham + l-cysteine and SAH + l-cysteine groups exhibited dramatic upregulation of CBS activity, whereas the SAH group showed low CBS activity (Figure 1C). Next, we measured H_2_S production in the PFC of the different groups because H_2_S production is an indirect measure of CBS activity (24). The l-cysteine-treated groups (Sham + l-cysteine and SAH + l-cysteine) produced more H_2_S than the Sham or SAH groups. Exposure to SAH slightly decreased H_2_S levels in the PFC, but the levels in the SAH group were not significantly different from those in the Sham group (Figure 1D). Co-treatment with AOAA suppressed the effects of l-cysteine on SAH.

### Administration of l-Cysteine Reduced Brain Edema and Improved Neurological Behavior at 48 h after SAH

**Figure 2:**
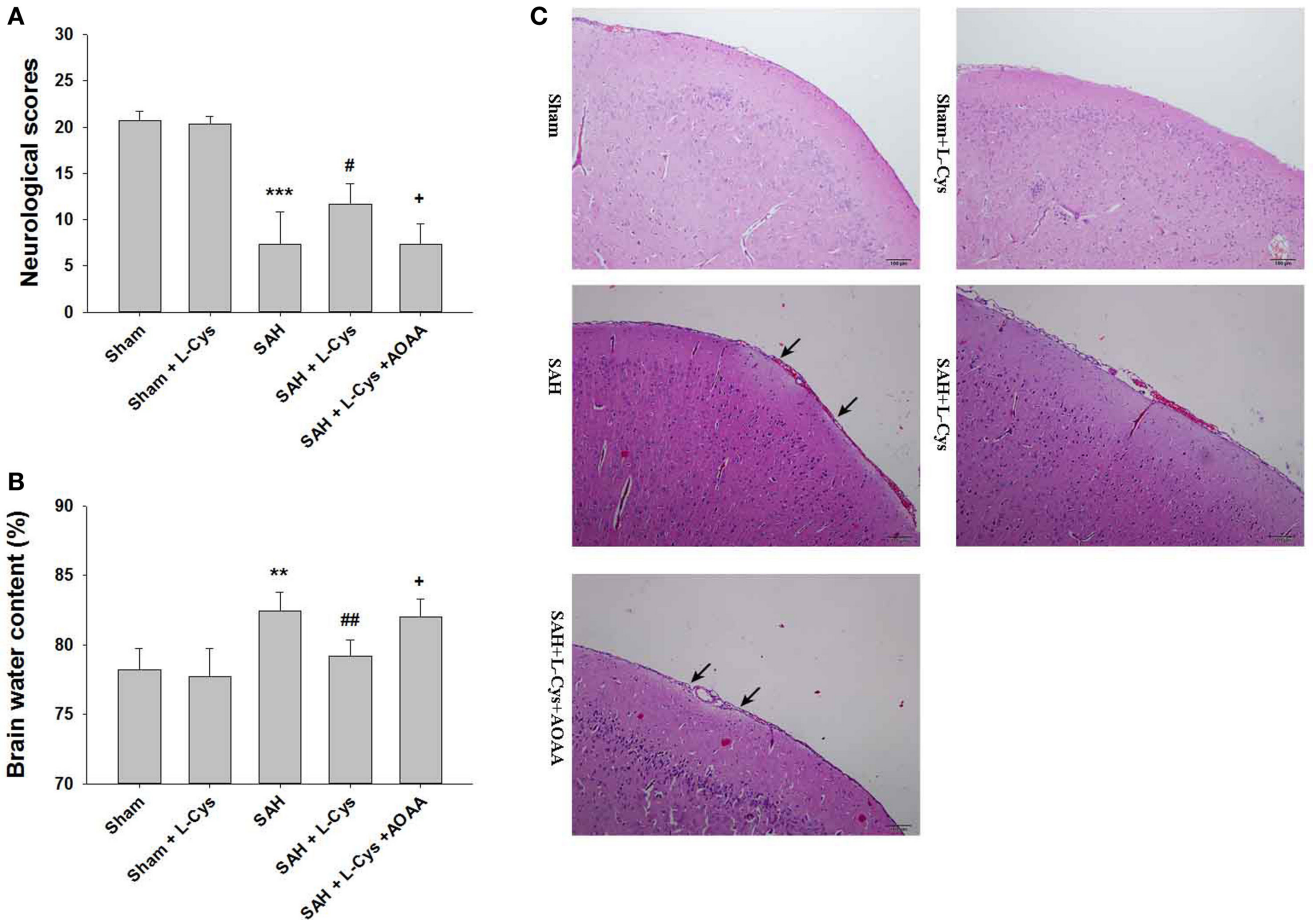
**l-Cysteine ameliorated subarachnoid hemorrhage (SAH)-induced brain injury**. **(A)** Neurological scores were recorded at 48 h after SAH (*n* = 6). **(B)** Brain water content of the cerebral cortex was measured at 48 h after SAH (*n* = 6). **(C)** H&E staining was performed on brain tissues at 48 h after SAH. Pathological changes included focal edema in the prefrontal cortex (black arrows indicated) (*n* = 4). Scale bar = 100 μm. The values represent the mean ± SD. **p < 0.01, ***p < 0.001 SAH vs Sham, ^#^*p* < 0.05, ^##^*p* < 0.01 SAH + l-Cys vs SAH, ^+^*p* < 0.05 SAH + l-Cys + AOAA vs SAH + l-Cys. AOAA, amino-oxyacetic acid.

Compared to the Sham group, the SAH groups had significantly lower neurological scores at 48 h (Figure 2A). l-Cysteine treatment improved neurological scores, but this treatment effect was reversed by AOAA (Figure 2A).

Brain edema (including both cerebral hemispheres) was evaluated immediately after the neurological assessment. The SAH-injured brain tissue had a significantly higher water content than that of the Sham and Sham + l-cysteine brain tissue. Post-SAH injection of l-cysteine reduced brain water content, but this outcome was reversed by AOAA administration (Figure 2B).

In the Sham and Sham + l-cysteine groups, the brain tissues had organized structural layers and cortical neurons with well-defined borders. However, in the SAH group, the cells were arranged sparsely, and the cell outline was fuzzy. Moreover, in the SAH group, we identified substantial edema in the PFC, which was pale in appearance, and shrunken neurons. l-Cysteine treatment ameliorated the edema and morphological damage induced by SAH (Figure 2C).

Additionally, a 5 mg/kg dose of AOAA did not induce further neuronal damage post-SAH (see Figure S1 in Supplementary Material).

### l-Cysteine Attenuated SAH-Induced Brain Injury by Reducing Neuronal Apoptosis

**Figure 3:**
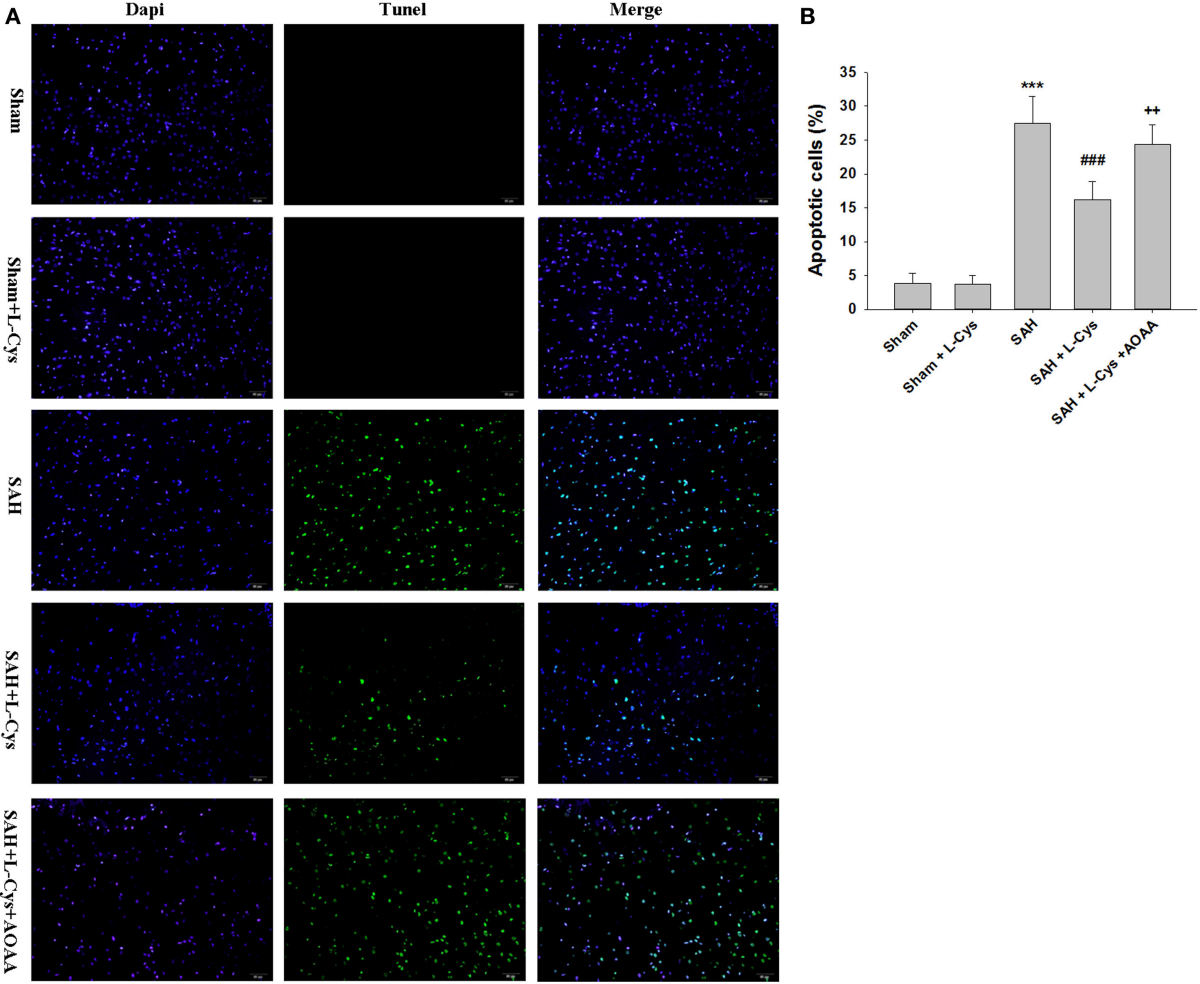
**l-Cysteine attenuates subarachnoid hemorrhage (SAH)-induced apoptosis**. **(A)** The detection of transferase dUTP nick end labeling (TUNEL)-positive cells in the prefrontal cortex was performed at 48 h after SAH. Scale bar = 50 μm. **(B)** Bar graphs showing the quantification of TUNEL-positive cells (*n* = 4). Scale bar = 50 μm. The values represent the means ± SD. ***p < 0.001 SAH vs Sham, ^###^*p* < 0.001 SAH + l-Cys vs SAH, ^++^*p* < 0.01 SAH + l-Cys + AOAA vs SAH + l-Cys. AOAA, amino-oxyacetic acid.

In the PFC of rats in the Sham and Sham + l-cysteine groups, TUNEL-positive cells were rarely detected, while in the SAH group, many TUNEL-positive cells were identified. The apoptosis in response to SAH was significantly ameliorated by treatment with l-cysteine, but this effect was inhibited by AOAA administration (Figure 3).

### l-Cysteine Inhibited SAH-Induced Caspase-3 Activation

**Figure 4:**
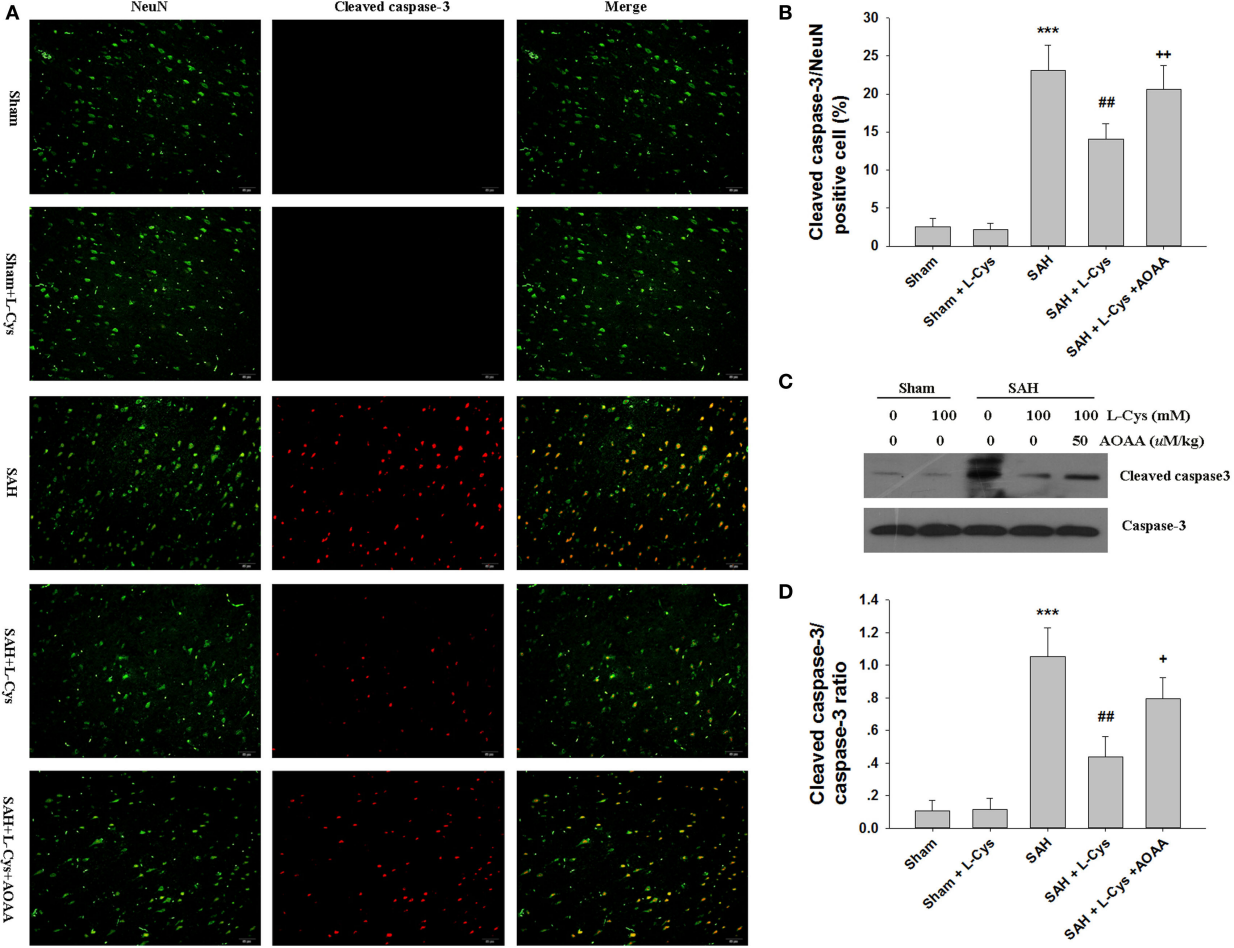
**The effect of l-cysteine on caspase-3 activation in subarachnoid hemorrhage (SAH)**. **(A)** Immunofluorescence staining revealed the colocalization of cleaved caspase-3 and NeuN in the prefrontal cortex at 48 h after SAH. Scale bar = 50 μm. **(B)** The bar graphs showing the quantification of cleaved caspase-3/NeuN-positive cells (*n* = 4). **(C)** The expression of cleaved caspase-3 was assessed using Western blot analysis. **(D)** Bar graphs showing the quantification of the protein levels of cleaved caspase-3 and caspase-3 were generated by Image-Pro Plus 6.0. The results are expressed as the cleaved caspase-3/caspase-3 ratio (*n* = 3). The values represent the mean ± SD. *p < 0.05, ***p < 0.001 SAH vs Sham, ^#^*p* < 0.05, ^###^*p* < 0.001 SAH + l-Cys vs SAH, ^+^*p* < 0.05, ^+++^*p* < 0.001 SAH + l-Cys + AOAA vs SAH + l-Cys. AOAA, amino-oxyacetic acid.

We used cleaved caspase-3/NeuN double staining to evaluate how l-cysteine treatment inhibited apoptosis after SAH. Few cleaved caspase-3-positive cells were detected in the Sham and Sham + l-cysteine groups, whereas numerous cleaved caspase-3/NeuN double-stained cells were seen in the SAH group (Figures 4A,B). l-Cysteine dramatically reduced cleaved caspase-3 expression levels, but this effect was blocked by AOAA. The effect of l-cysteine on SAH-induced caspase-3 activation was confirmed by Western blot (Figures 4C,D).

### l-Cysteine Restored Bcl-2 and Bax Expression Levels after SAH

**Figure 5:**
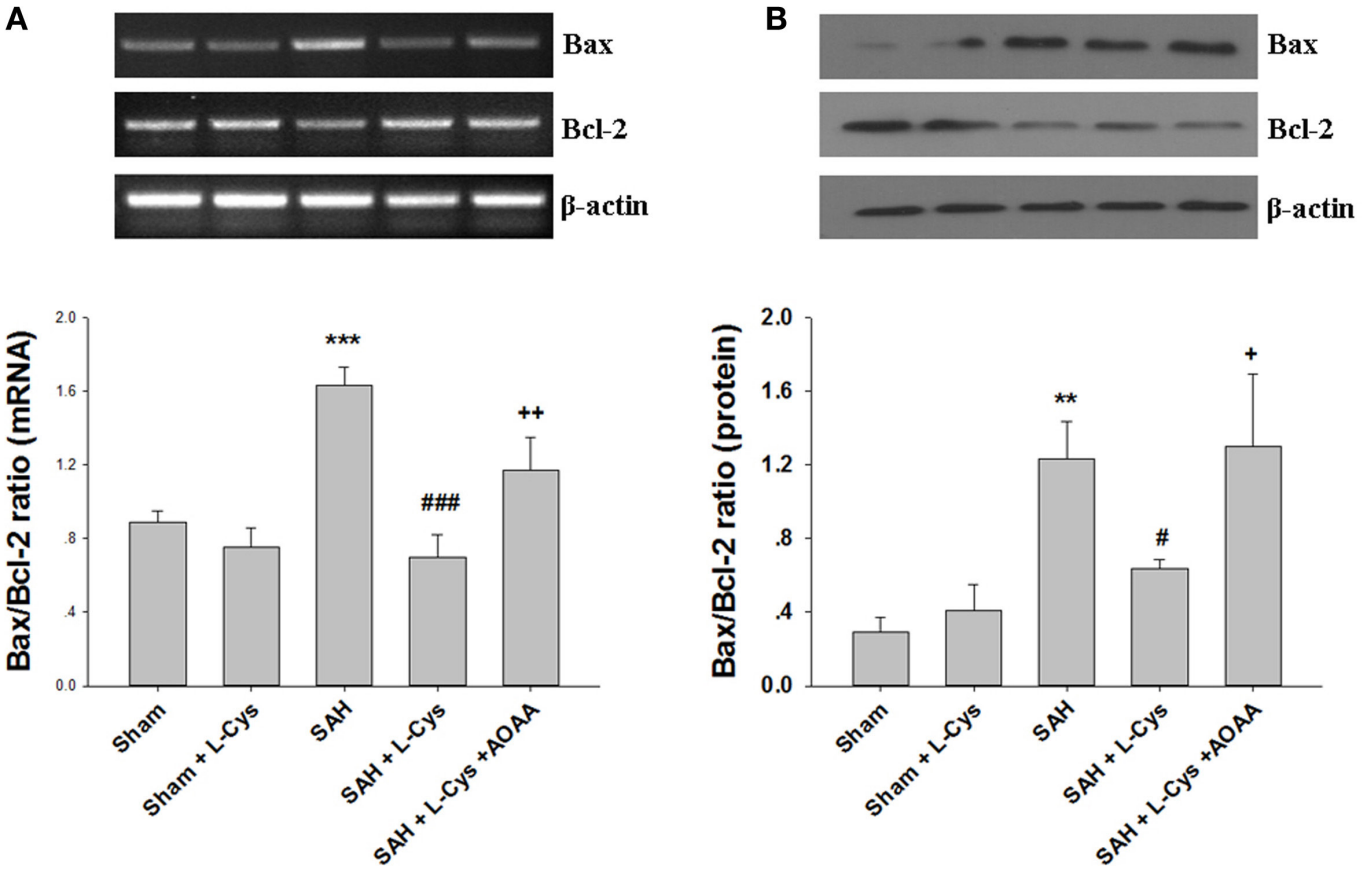
**Effects of l-cysteine on Bax and Bcl-2 at the mRNA and protein levels**. **(A)** The relative expression levels of Bax and Bcl-2 mRNA in the prefrontal cortex (PFC) were analyzed by semi-quantitative reverse transcription polymerase chain reaction. The densities of the protein bands were analyzed and normalized to β-actin (*n* = 3). **(B)** Representative Western blots showing the levels of Bax and Bcl-2 in the PFC and bar graphs showing the quantification of the protein levels of Bax and Bcl-2 (*n* = 3). The mRNA and protein levels were obtained from three independent experiments. The values represent the means ± SD. **p < 0.01, ***p < 0.001 subarachnoid hemorrhage (SAH) vs Sham, ^#^*p* < 0.05, ^###^*p* < 0.001 SAH + l-Cys vs SAH, ^+^*p* < 0.05, ^++^*p* < 0.01 SAH + l-Cys + AOAA vs SAH + l-Cys. AOAA, amino-oxyacetic acid.

Because Bcl-2 and Bax are key regulators of the mitochondrial apoptosis pathway in cells, we evaluated the expression levels of Bcl-2 and Bax at both the mRNA and protein levels. As shown in Figure 5, SAH markedly increased the Bax/Bcl-2 ratio at the mRNA and protein levels at 48 h after injury. However, the increased Bax/Bcl-2 ratio was reduced by treatment with l-cysteine. The effect of l-cysteine on the SAH-induced elevation in the Bax/Bcl-2 ratio was reversed by AOAA (Figures 5A,B).

### l-Cysteine Increased the Expression of BDNF Following SAH-Induced Injury

**Figure 6:**
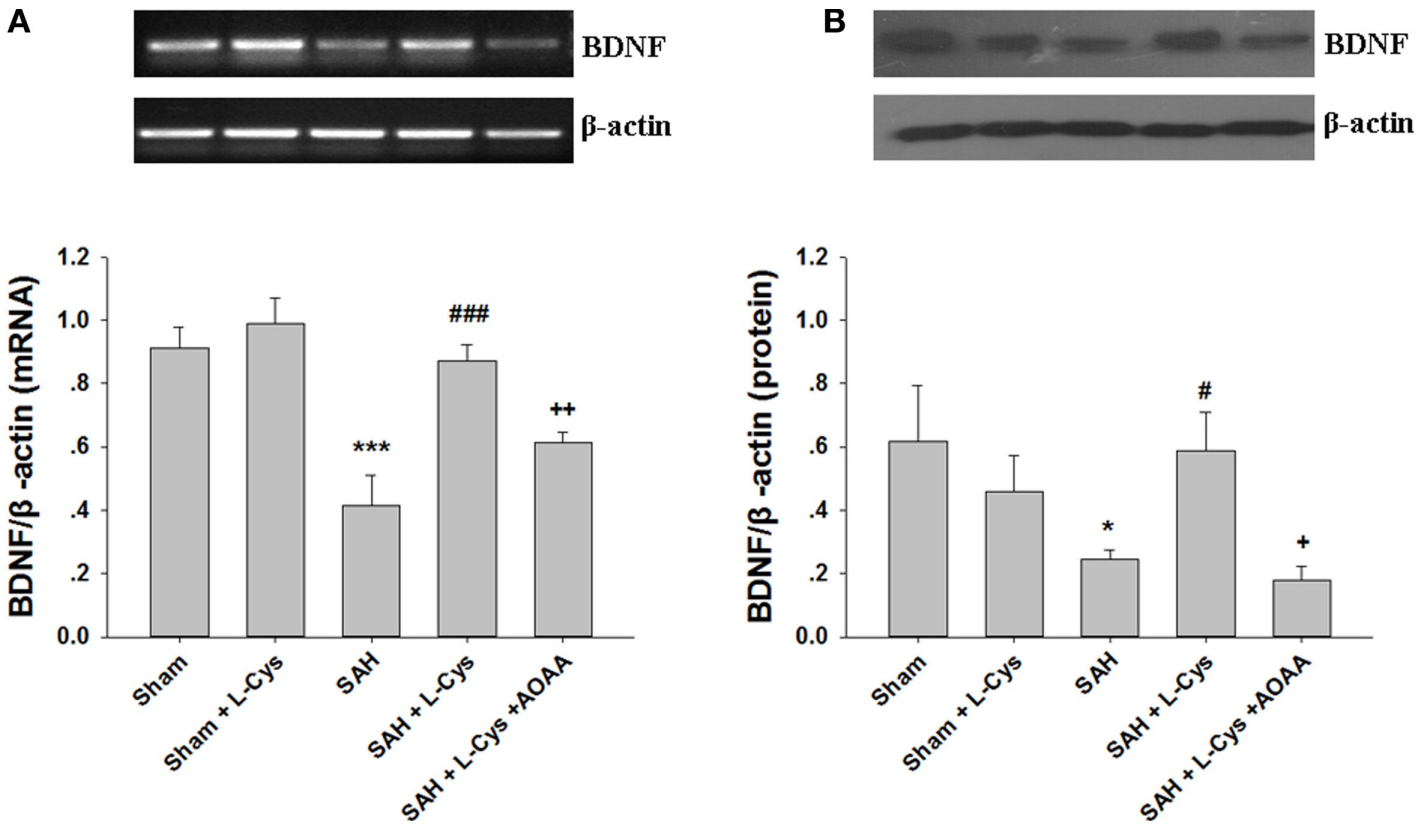
**The effect of l-cysteine on brain-derived neurotrophic factor (BDNF) expression levels at the mRNA and protein levels**. **(A)** The BDNF expression levels at the mRNA level in the prefrontal cortex was assessed by semi-quantitative reverse transcription polymerase chain reaction at 48 h after subarachnoid hemorrhage (SAH). Each value was normalized to β-actin. Bar graphs showing the quantification of the BDNF mRNA levels were generated by Image-Pro Plus 6.0 (*n* = 4). **(B)** The BDNF protein expression level was analyzed by Western blotting at 48 h after SAH, and β-actin was used to evaluate protein loading. The bar graphs showing the quantification of the protein levels of BDNF were generated by Image-Pro Plus 6.0 (*n* = 3). The values represent the mean ± SD. *p < 0.05, ***p < 0.001 SAH vs Sham, ^#^*p* < 0.05, ^###^*p* < 0.001 SAH + l-Cys vs SAH, ^+^*p* < 0.05, ^++^*p* < 0.01 SAH + l-Cys + AOAA vs SAH + l-Cys. AOAA, amino-oxyacetic acid.

To determine whether l-cysteine can affect production of neuroprotective factors, the BDNF concentration of the PFC was measured at 48 h after SAH. As shown in Figure 6A, the BDNF mRNA expression level was significantly lower at 48 h in the SAH group than that in the Sham group. l-Cysteine significantly increased the expression level of BDNF mRNA in the PFC 48 h post-SAH exposure (Figure 6A). Consistent with the changes in the mRNA, the SAH-induced decrease in the BDNF protein levels was also reversed by l-cysteine treatment, but the effect of l-cysteine was reversed by AOAA (Figure 6B).

### Administration of l-Cysteine Improves CREB phosphorylation *In Vivo*

**Figure 7:**
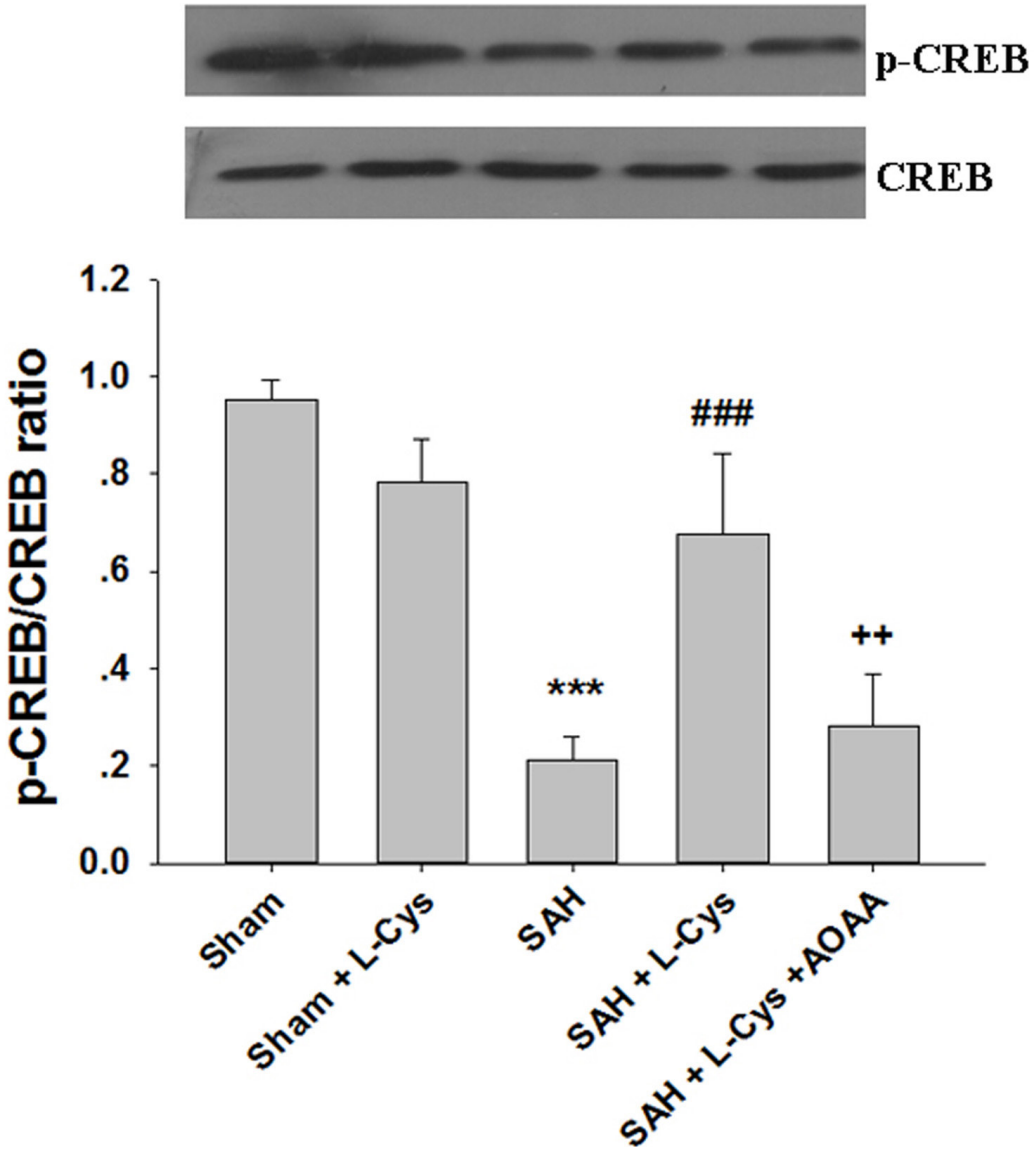
**Effects of l-cysteine on CREB phosphorylation after subarachnoid hemorrhage (SAH)**. At 48 h after SAH, whole prefrontal cortex extracts were subjected to Western blot analysis using antibodies against phospho-cAMP response element binding protein (p-CREB) and CREB. Bar graphs showing the quantification of the expression levels of p-CREB/CREB were generated by Image-Pro Plus 6.0 (*n* = 3). The values represent the means ± SD. ***p < 0.001 SAH vs Sham, ^###^*p* < 0.001 SAH + l-Cys vs SAH, ^++^*p* < 0.001 SAH + l-Cys + AOAA vs SAH + l-Cys. AOAA, amino-oxyacetic acid.

Phosphorylated CREB regulates the transcription of several genes that code for molecules involved in neuronal plasticity, including BDNF, tyrosine hydroxylase, and neural cell adhesion molecule; these molecules are associated with the stress response. Thus, we examined the CREB phosphorylation levels after SAH and l-cysteine treatment. As shown in Figure 7, phosphorylated CREB expression significantly decreased at 48 h after SAH compared to that in the Sham group. Treatment with l-cysteine significantly increased the expression level of phosphorylated CREB in the PFC at 48 h post-SAH exposure. Additionally, the effect of l-cysteine on the SAH-induced CREB phosphorylation levels was reversed by AOAA.

### Effects of l-Cysteine on Synaptic Structure and Expression of Synaptophysin and PSD95 after SAH

**Figure 8:**
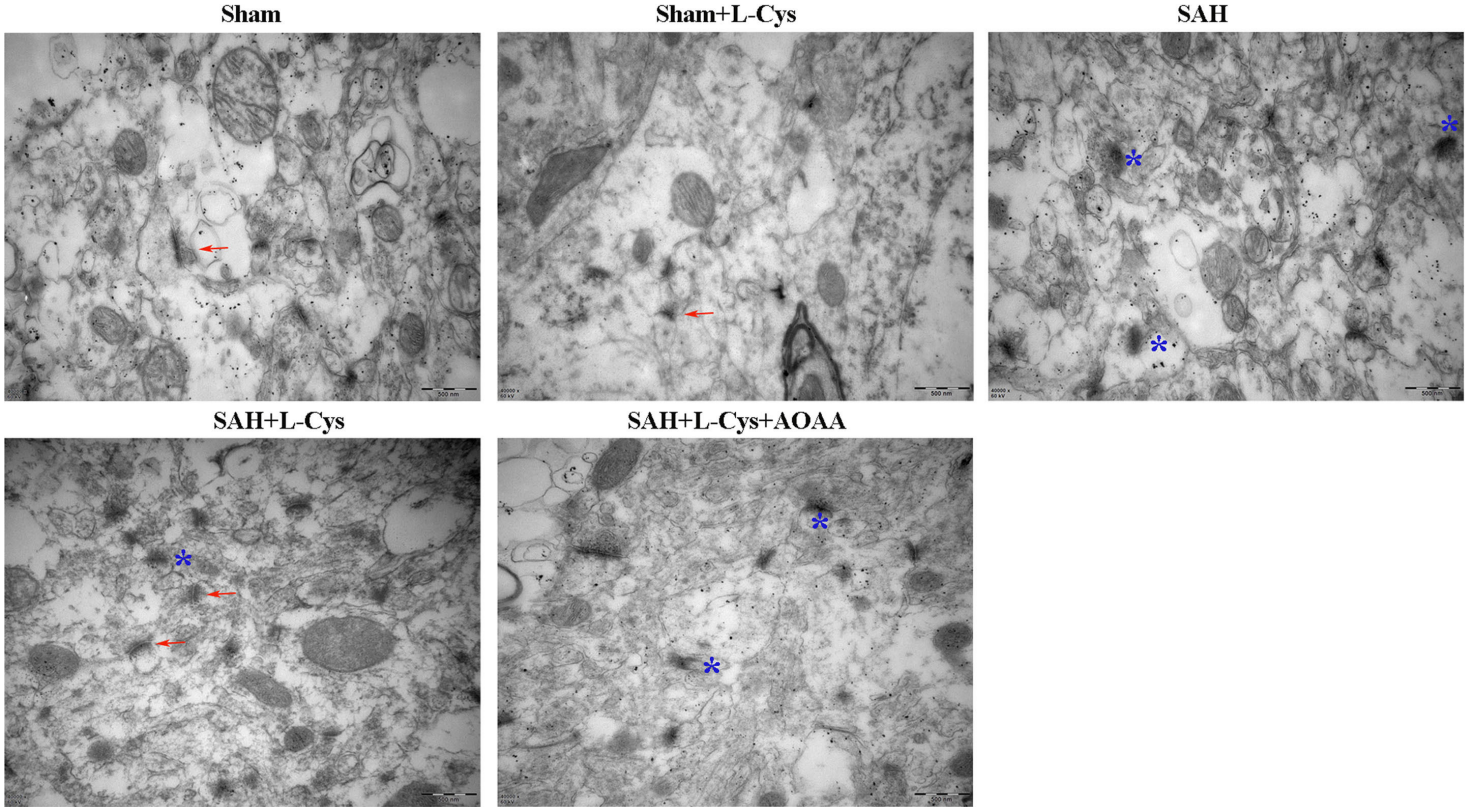
**Effects of l-cysteine treatment on synaptic changes in the prefrontal cortex (PFC)**. Representative transmission electron microscopy images of the PFC from each group. The arrows indicate regular synaptic structures. The stars denote collapsed synapses. Scale bar = 500 nm.

Neuronal damage, including synapse collapse, occurs after SAH; therefore, we investigated the morphological changes in the synapses of the PFC using TEM. Compared with the Sham and Sham + l-cysteine groups (Figure 8), the SAH group exhibited vague structural changes in the synapses, which included swollen borders and dark staining that indicated degeneration. In addition, the number of normal synapses decreased in the SAH group. l-cysteine treatment dramatically ameliorated the synaptic damage and upregulated the number of synapses in the SAH group, whereas the effects of l-cysteine were abrogated by AOAA.

**Figure 9:**
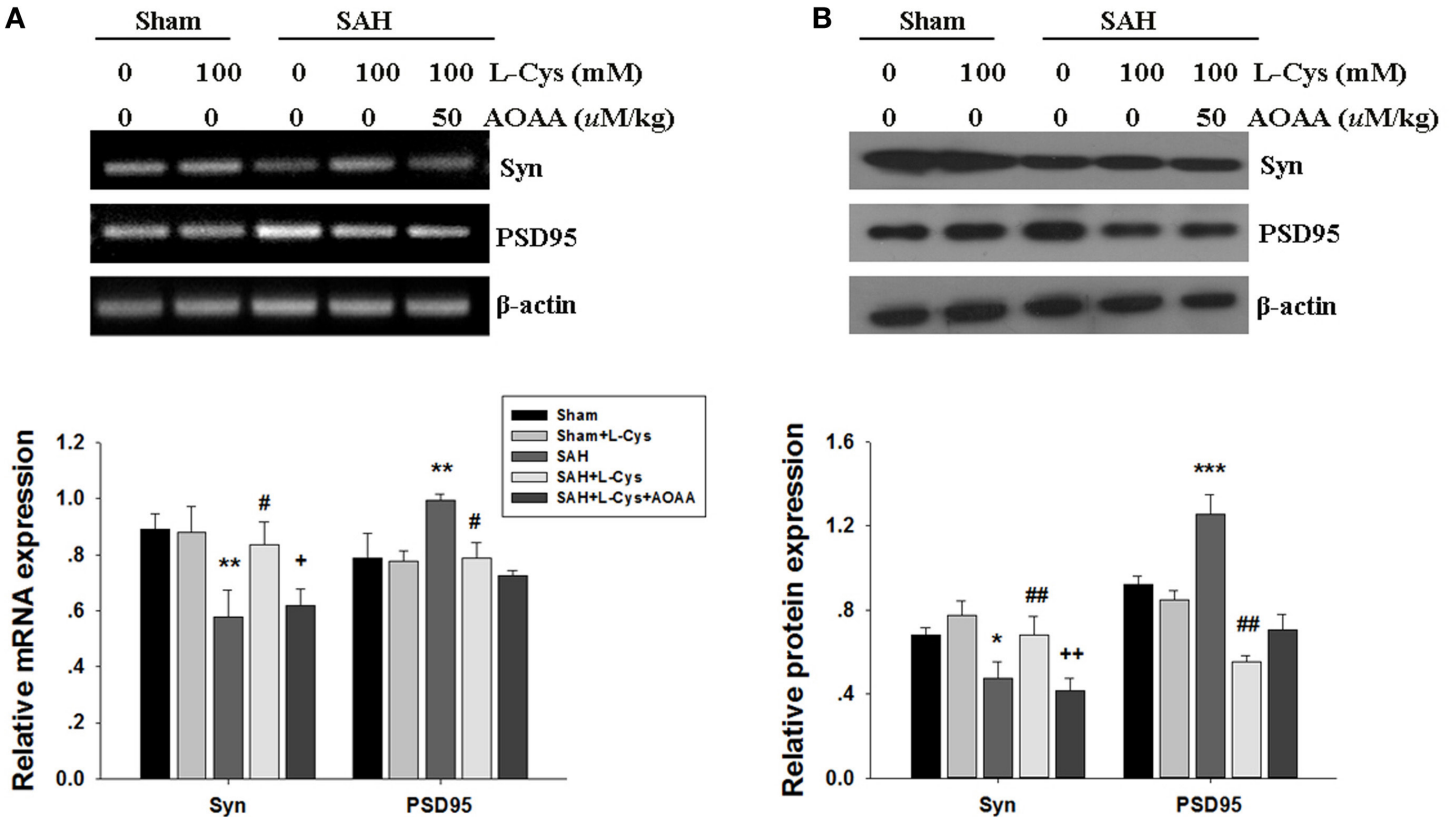
**Effects of l-cysteine treatment on synaptophysin and PSD95 expression levels in the prefrontal cortex (PFC)**. **(A)** The mRNA levels of synaptophysin and PSD95 were measured by semi-quantitative reverse transcription polymerase chain reaction. Each value was normalized to β-actin. Bar graphs showing the quantification of the mRNA levels of synaptophysin and PSD95 were generated by Image-Pro Plus 6.0 (*n* = 3). **(B)** At 48 h after subarachnoid hemorrhage (SAH), whole PFC extracts were subjected to Western blot analysis using antibodies against synaptophysin and PSD95. The bar graphs showing the quantification of the mRNA levels of synaptophysin and PSD95 were generated by Image-Pro Plus 6.0 (*n* = 3). The values represent the mean ± SD of three independent experiments. *p < 0.05, **p < 0.01, ***p < 0.001 SAH vs Sham, ^#^*p* < 0.05, ^##^*p* < 0.01 SAH + l-Cys vs SAH, ^+^*p* < 0.05 SAH + l-Cys + AOAA vs SAH + l-Cys. AOAA, amino-oxyacetic acid.

Next, we measured the level of the presynaptic marker synaptophysin and the postsynaptic marker PSD95. Synaptophysin was significantly decreased at both the mRNA and protein levels in the SAH group. Treatment with l-cysteine significantly increased the expression of synaptophysin in the PFC at 48 h post-SAH exposure. However, compared with the Sham group, the mRNA and protein levels of PSD95 were significantly increased at 48 h in the SAH group (Figures 9A,B). l-cysteine significantly decreased the expression of PSD95 in the PFC at 48 h post-SAH exposure. AOAA reversed the effects of l-cysteine on the synaptophysin and PSD95 expression levels.

## Discussion

In the current research, we demonstrated that l-cysteine could enhance H_2_S levels in the brain *via* an interaction with CBS; additionally, l-cysteine played a neuroprotective role in SAH by ameliorating cerebral edema and neuronal apoptosis. Moreover, l-cysteine sensitized the CREB–BDNF pathway and upregulated the expression of proteins related to synaptic plasticity. The positive effects initiated by l-cysteine were significantly abrogated when the CBS antagonist AOAA was administered.

Cystathionine-β-synthase is a pyridoxal-5′-phosphate-dependent enzyme that catalyzes β-replacement in which the β-position of the substrate is substituted by a nucleophile YH (25) and transforms substrates to 2-mercaptoethanol and H_2_S (26, 27). A close association between mutations in several regions of the human CBS gene and mental disorders and vascular diseases has been identified (28). In the rat brain, CBS was more highly expressed than cystathionine γ lyase and was mainly responsible for H_2_S generation (7). l-cysteine, an amino acid containing the electronegative substituent -SH, is the preferred substrate for H_2_S, which accounts for 70% of H_2_S production (29). Li et al. demonstrated that l-cysteine administration upregulated the production of H_2_S, whereas AOAA markedly attenuated the effects of l-cysteine in a dose-dependent manner (30). In Kmamt’s study, administration of NaHS, a, H_2_S donor, reversed the decreased expression of CBS, an H_2_S-metabolizing enzyme (31). In our study, l-cysteine was first administered to the animals *via* intracerebroventricular injection at 30 min post-SAH and increased CBS activity and expression in the PFC, which is consistent with the H_2_S production levels. We suggest that l-cysteine provokes a potential feedback loop to increase CBS activity in response to SAH and increase H_2_S production to exert neuroprotective effects.

Cell apoptosis is a major characteristic of EBI, and the mitochondrial pathway may also be involved (32). Additionally, exogenous H_2_S was recently shown to protect against global and focal cerebral ischemia/reperfusion injury (33, 34). However, whether H_2_S can preserve neurons through l-cysteine metabolism after SAH remains unknown. In our study, we observed numerous TUNEL-positive cells in the CNS of the SAH group, which is consistent with Chen’s report (35). l-cysteine administration can reduce the number of apoptotic cells induced by SAH. Meanwhile, we further investigated the Bax/Bcl-2 ratio. The Bcl-2 family member Bax was markedly upregulated after SAH, which resulted in the release of cytochrome *c* to the cytosol (32). We found that the SAH-induced upregulation of the Bax/Bcl-2 ratio could be reversed by l-cysteine administration. Moreover, we analyzed the activation of caspase-3; cytochrome *c* release triggers the cleavage of the caspase-3 protein, which results in DNA fragmentation and apoptosis (36). Inhibition of cleaved caspase-3 could reduce neuronal loss in SAH models (37). Our data revealed that treatment with l-cysteine prevented the SAH-induced increase in cleaved caspase-3 in the PFC. Our findings suggest that l-cysteine could protect neurons from apoptosis after SAH. However, when AOAA was administered with l-cysteine, all the beneficial effects on apoptosis were abolished. Thus, we hypothesize that the neuroprotective effects of l-cysteine on SAH may be due to an increase in endogenous H_2_S.

Brain-derived neurotrophic factor is a growth factor and supports neuronal survival, plasticity and neurogenesis (38, 39). Moreover, BDNF is involved in the pathophysiology of SAH. For example, clinical evidence has shown that a BDNF polymorphism is associated with poor patient recovery from SAH (40, 41). Animal experiments have demonstrated that exogenous BDNF infusion or upregulation of its expression improves neurobehavioral outcomes after SAH (42, 43). Regarding the underlying mechanisms of the neuroprotective effect of BDNF on neuronal apoptosis, some studies have shown that these effects are dependent on the activation of the PI3K/Akt and/or ERK signaling cascade, which subsequently activates CREB phosphorylation and promotes neuronal survival (44, 45). Previous studies have reported that H_2_S promotes BDNF expression, and blocking the BDNF-TrkB pathway reverses the H_2_S-mediated neuroprotection against apoptosis and oxidative stress in neurons (46, 47). Moreover, H_2_S can activate the CREB signaling pathway and prevent ischemia-reperfusion injury in the brain (48). In our study, the expression levels of p-CREB and BDNF increased after l-cysteine administration, which suggests that H_2_S could activate the CREB signaling pathway and increase the expression of its downstream pro-survival gene, BDNF. Importantly, these findings raise the possibility that H_2_S exerts anti-apoptotic effects *via* upregulation of p-CREB and BDNF in the PFC. Considering that AOAA blocks CBS, an l-cysteine catalyst, and reduces H_2_S production, we hypothesize that l-cysteine simulates CREB–BDNF expression in the CNS *via* inducing H_2_S during SAH.

Recently, Shen et al. reported that neuronal damage, including synapse collapse, occurs after SAH (49). Synapses are critical structural units for transmitting information in the brain. Accumulating evidence has demonstrated that changes in synapse density are highly correlated with cognitive status (50, 51). Synaptophysin and PSD95 are reliable markers to indirectly evaluate the integrality and function of the synapses (52, 53). Synaptophysin is a marker of the presynaptic nerve terminal density, which is essential for vesicle fusion and the release of neurotransmitter (54). The decrease in synaptophysin in CNS diseases indicates a reduction in synaptic plasticity (55). PSD95 is a scaffold protein that anchors and organizes NMDA receptors and controls the number and size of dendritic spines (56). We demonstrated that injection of l-cysteine after SAH significantly attenuates synaptic damage by ameliorating structural degeneration and upregulating the number of healthy synapses. At the mRNA and protein levels, a decrease in synaptophysin occurs in SAH, and synaptophysin levels are improved by l-cysteine administration, which indicates the potential role of H_2_S in stimulating changes in synaptophysin levels. To our surprise, we observed that PSD95 was upregulated after SAH; in contrast, previous studies have shown that PSD95 expression is decreased in diverse brain diseases (57, 58). We additionally showed that l-cysteine could suppress PSD95 expression. We postulate that interactions between PSD95 and the NMDA receptor are increased after SAH, which leads to neuronal injury. However, AOAA did not block the effect of l-cysteine on PSD95 expression in SAH. The improvement induced by l-cysteine may not be achieved through the regulation of PSD95 by H_2_S as we expected.

There are several limitations to our study. First, we used AOAA to block CBS activity and l-cysteine function, but the CNS contains other enzymes that can produce H_2_S, such as 3-mercaptopyruvate sulfurtransferase, which need to be investigated in future studies (6). Second, although apoptosis is a major contributor to EBI, other factors, including cerebral vasospasm, inflammation and oxidative stress, may also be responsible for the development of EBI in SAH (2, 59). Determining whether l-cysteine has a beneficial effect on these factors and exploring the potential underlying mechanism of its effects will require further study. Third, how l-cysteine regulates the expression of synaptophysin and PSD95 and how these factors affect neurological function were not determined (31, 60). Finally, the route, timing and dosage of l-cysteine treatment need to be further elucidated.

In summary, l-cysteine treatment could alleviate the development of EBI induced by SAH through multiple mechanisms, including reducing cell apoptosis, upregulating BDNF–CREB expression, and improving synapse density, by activating the CBS/H_2_S system.

## Ethics Statement

The International Guiding Principles for Animal Research, as stipulated by the CIOMS and adopted by the Laboratory Animal Center at Shandong University, were generally followed for all animal handling and care. Researchers handling animals were systematically trained following the Institutional Animal Care and Use Committee (IACUC) guidebook. Euthanasia for animal models was performed as instructed under “AVMA Guidelines for the Euthanasia of Animals: 2013 Edition.” All efforts were made to reduce the number of animals used and their suffering, in compliance with 3R principles. The protocol was approved by the Ethic Committee of Qilu Hospital.

## Author Contributions

GL and ZW were involved in designing the study, interpreting the data and writing the manuscript; TL and LXW performed the majority of the experiments and contributed to the analysis of the data; QH, SL, XMB, and YKX were responsible for the animal model; TTZ and SSB were responsible for Western blotting; and XQG and SHW were responsible for preparing pathological sections. The authors have no conflicts of interest to declare.

## Conflict of Interest Statement

The authors declare that the research was conducted in the absence of any commercial or financial relationships that could be construed as a potential conflict of interest.

## Abbreviations

AOAA, amino-oxyacetic acid; BDNF, brain-derived neurotrophic factor; CBS, cystathionine-β-synthase; CNS, central nervous system; CREB, cAMP response element binding protein; p-CREB, phospho-cAMP response element binding protein; DAB, diaminobenzidine; DAPI, 4′,6-diamidino-2-phenylindole dihydrochloride; EBI, early brain injury; H_2_S, hydrogen sulfide; IHC, immunohistochemical staining; NNDPD, *N*,*N*-dimethyl-*p*-phenylenediamine sulfate; SAH, subarachnoid hemorrhage; TEM, transmission electron microscopy.
