# Supplementary material for: Neuroprotective Roles of l-Cysteine in Attenuating Early Brain Injury and Improving Synaptic Density via the CBS/H2S Pathway Following Subarachnoid Hemorrhage in Rats
Source: Front Neurol. 2017 May 2;8:176. doi: 10.3389/fneur.2017.00176 (PMC5411453; doi:10.3389/fneur.2017.00176)
Supplement: Supplementary file 1 [file Table_1.DOC]

[**Table 1**](http://www.ncbi.nlm.nih.gov/pmc/articles/PMC2013981/table/tbl1/) PCR primers used in this study

| *Gene* | *Forword (*5’→3’*)* | *Reverse (*5’→3’*)* |
| --- | --- | --- |
| Bax | GGT TGC CCT CTT CTA CTT TGC | TCT TCC AGA TGG TGA GCG AG |
| Bcl-2 | GGA TGA CTT CTC TCG TCG CTA C | TGA CAT CTC CCT GTT GAC GCT |
| BDNF | AGC TGA GCG TGT GTG ACA GT | ACC CAT GGG ATT ACA CTT GG |
| Synaptophsin | CAAGAAATACCGCTACCAAGATG | CCCTCTGTTCCATTCACCTG |
| PSD95 | ATGGCACGTAATGGAGACTAC | TCTTGTGTAGTCGAACCATCTG |
| β -actin | CTA TTG GCA ACG AGC GGT TCC | CAG CAC TGT GTT GGC ATA GAG G |
