# Supplementary material for: Neuroprotective Roles of l-Cysteine in Attenuating Early Brain Injury and Improving Synaptic Density via the CBS/H2S Pathway Following Subarachnoid Hemorrhage in Rats
Source: Front Neurol. 2017 May 2;8:176. doi: 10.3389/fneur.2017.00176 (PMC5411453; doi:10.3389/fneur.2017.00176)
Supplement: Supplementary file 2 [file Data_Sheet_1.DOCX]

**
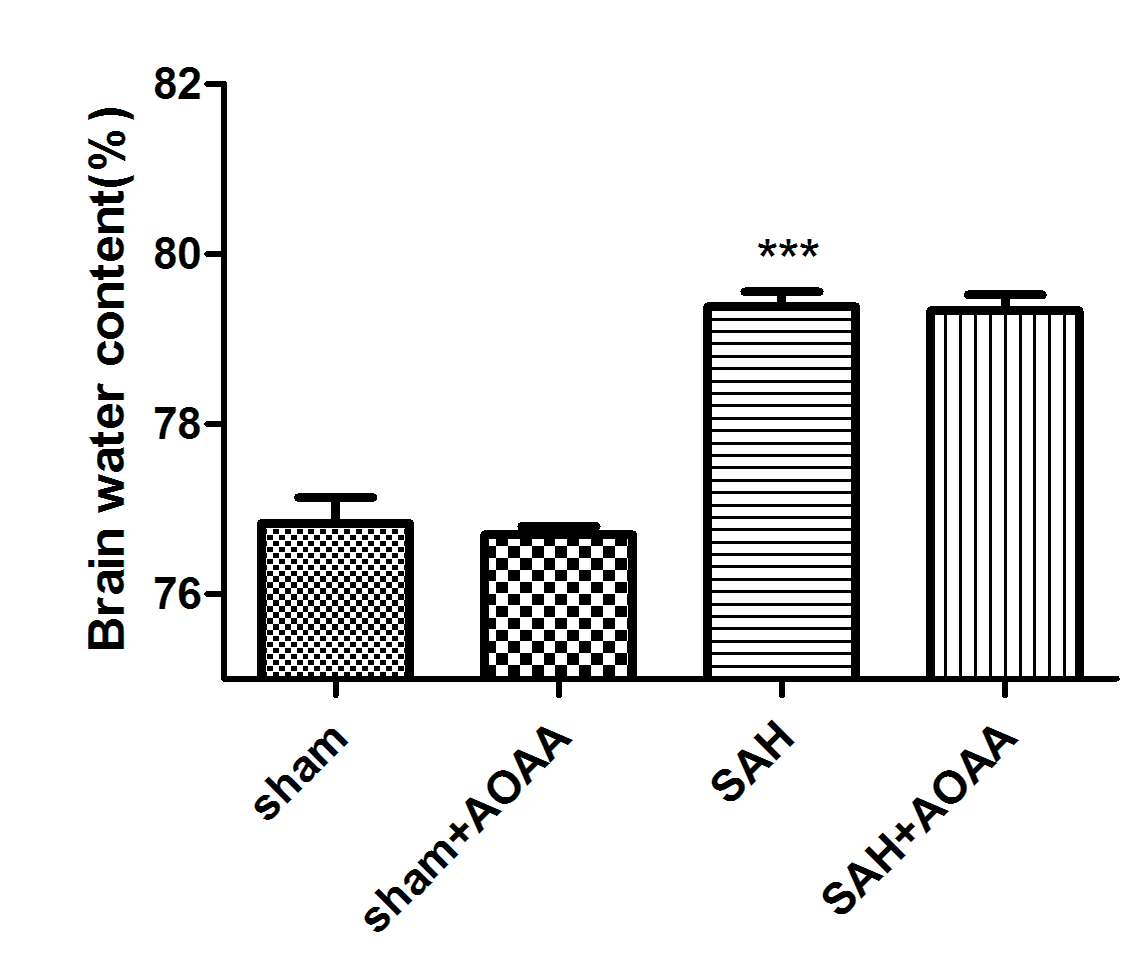
**

**Fig 1. Effects of AOAA on brain edema following SAH**

Brain water content was measured 48h post SAH insult. Values represent the mean ± SD. n=3.*** p <0.001 SAH VS Sham.


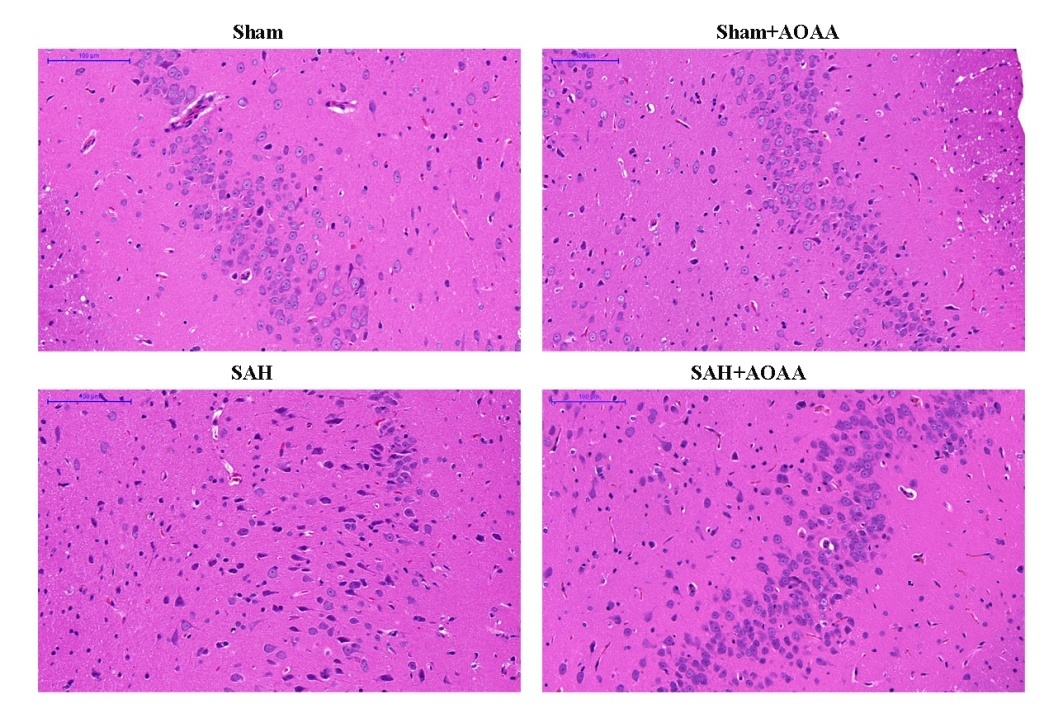


Fig 2. Effects of AOAA on brain injury after SAH

HE staining was operated and observed at 48h after SAH. Pathological change indicates cell death and degeneration in PFC.n=3. Scale bar=100μm.
